# Supplementary material for: Human agency beliefs influence behaviour during virtual social interactions
Source: PeerJ. 2017 Sep 20;5:e3819. doi: 10.7717/peerj.3819 (PMC5610555; doi:10.7717/peerj.3819)
Supplement: Supplemental Information 1 [file peerj-05-3819-s001.pdf]

Supplementary Material 1.

Task instructions cards.

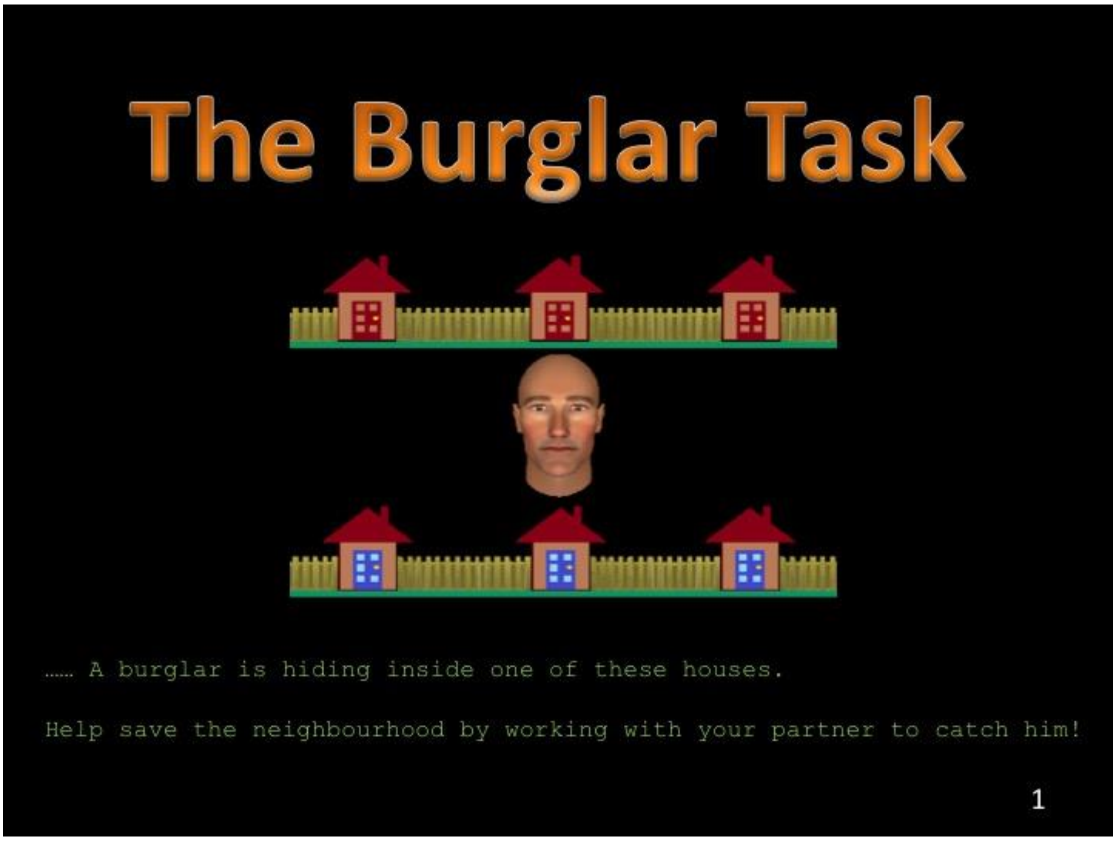

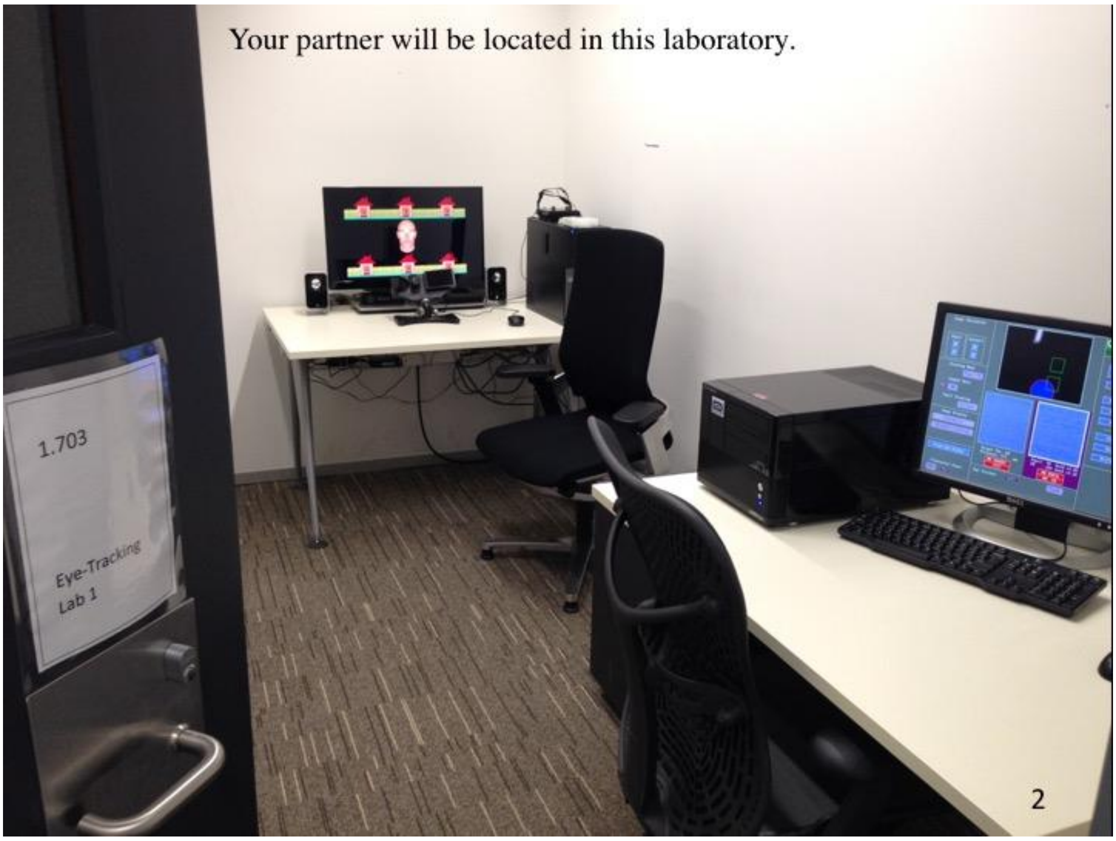

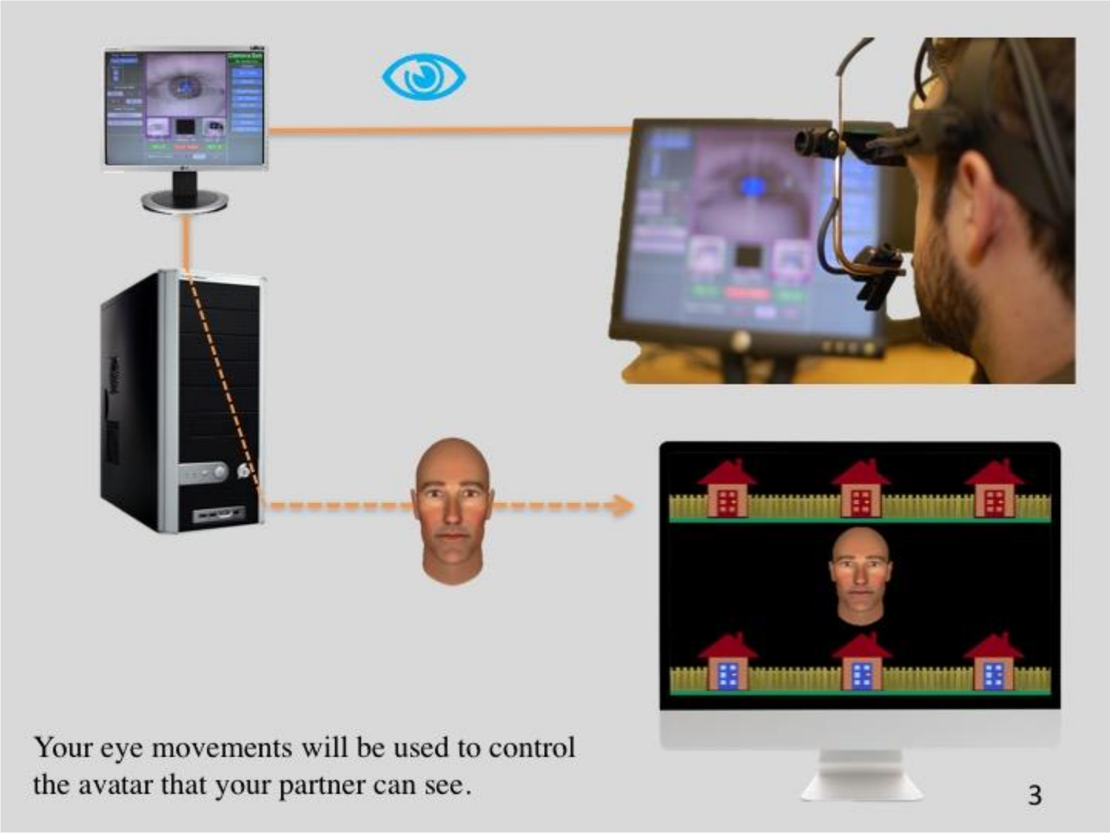
Your eye movements will be used to control
the avatar that your partner can see.
3

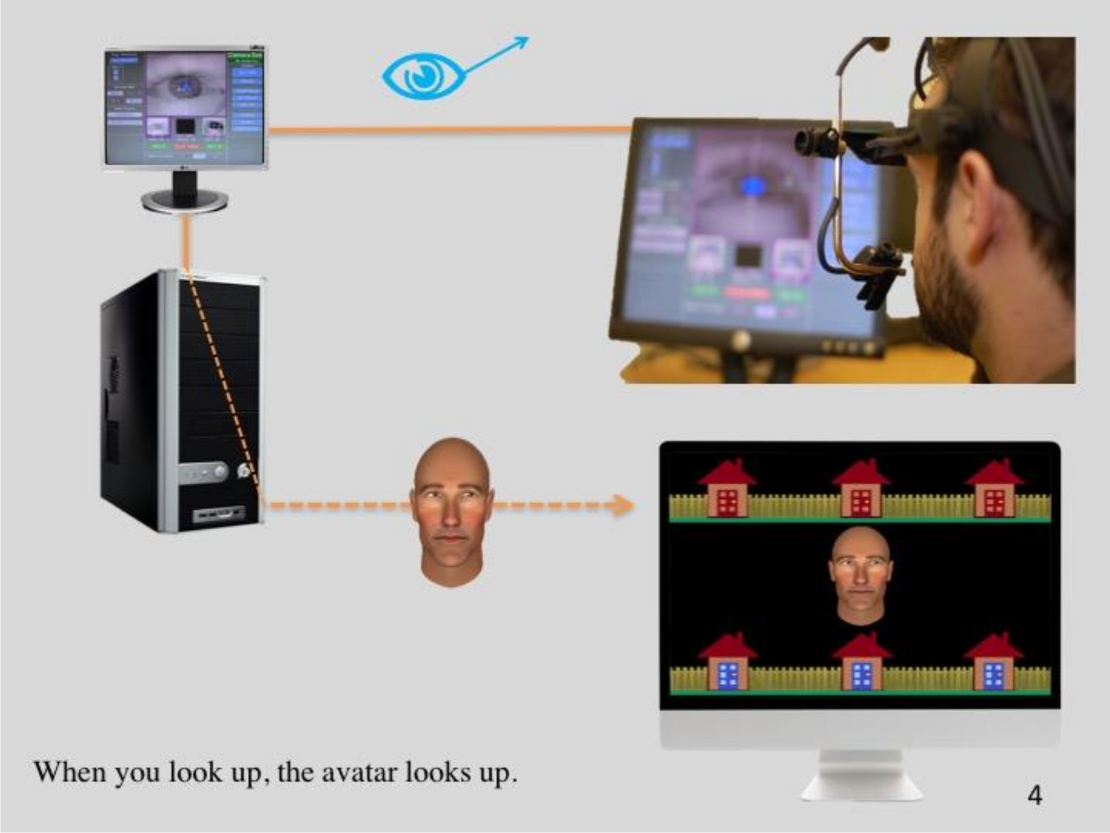
When you look up, the avatar looks up.
4

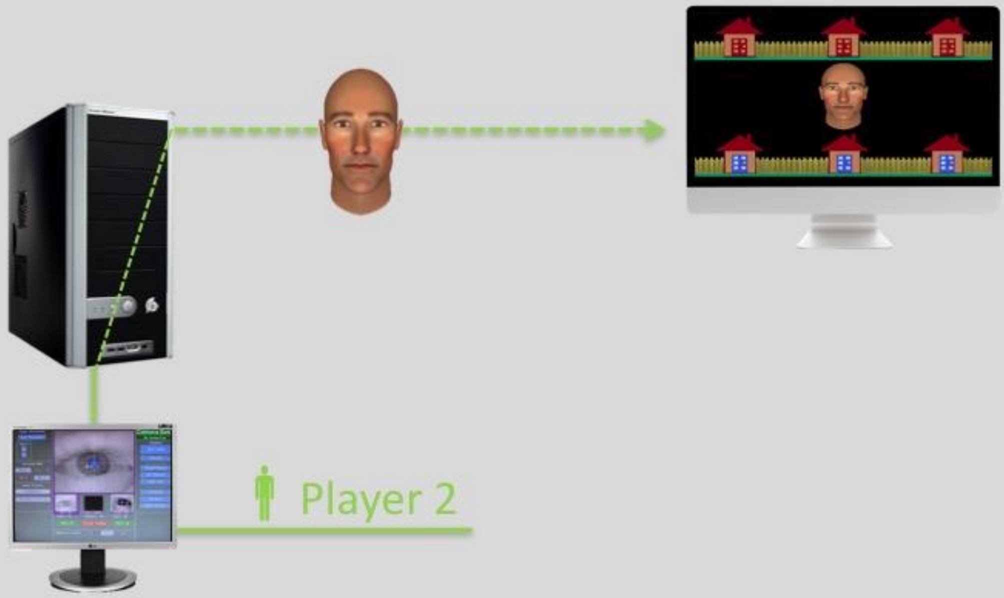

In the same way, your partner will control the avatar that you can see. 5

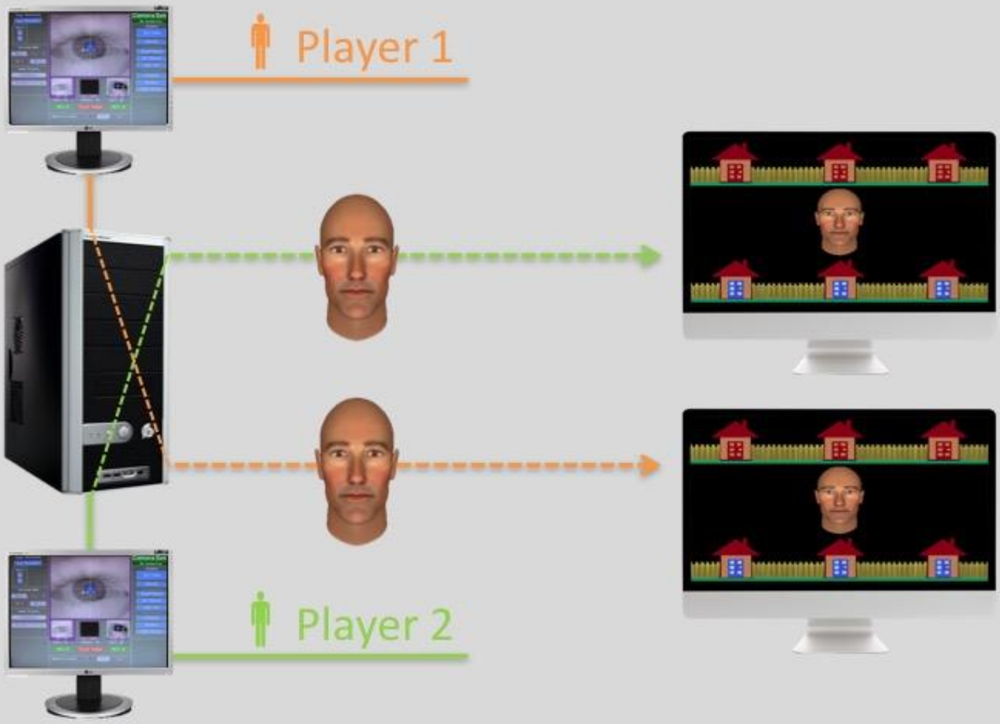

Your partner will see your eye movements.
You will see your partner's eye movements. 6

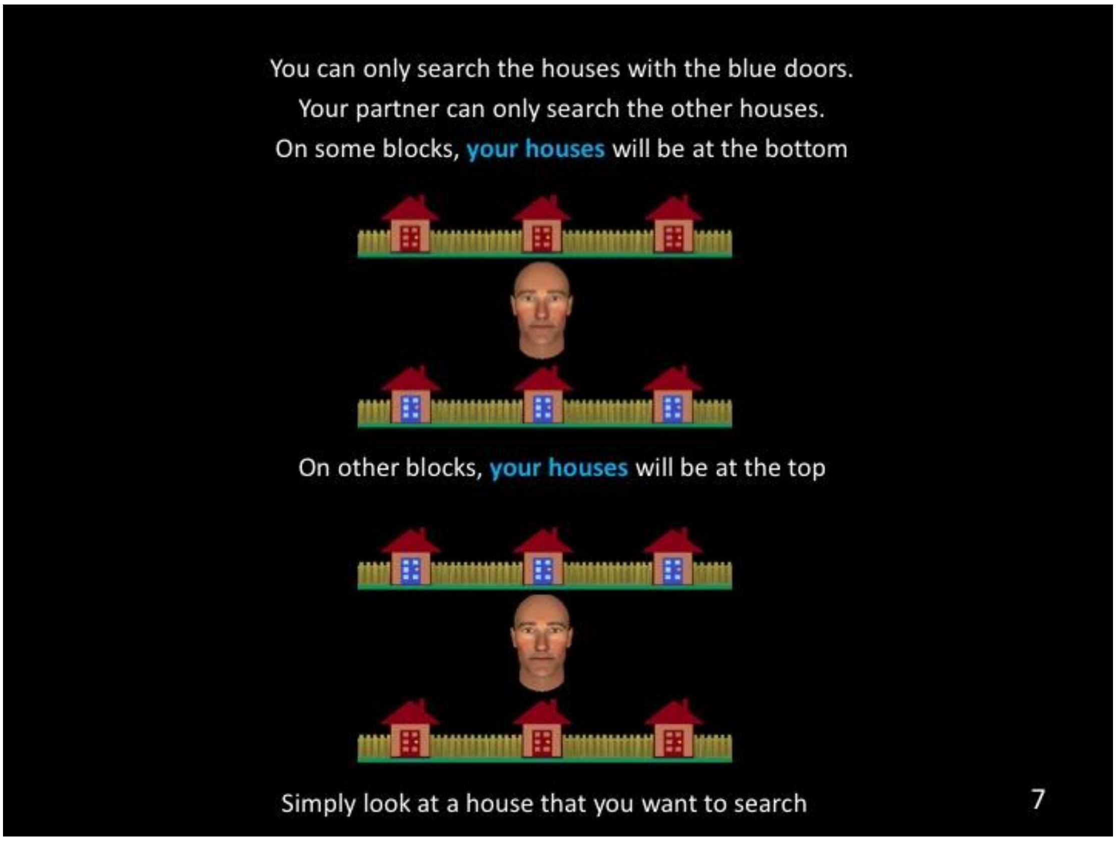
You can only search the houses with the blue doors.
Your partner can only search the other houses.
On some blocks, your houses will be at the bottom
On other blocks, your houses will be at the top
Simply look at a house that you want to search
7

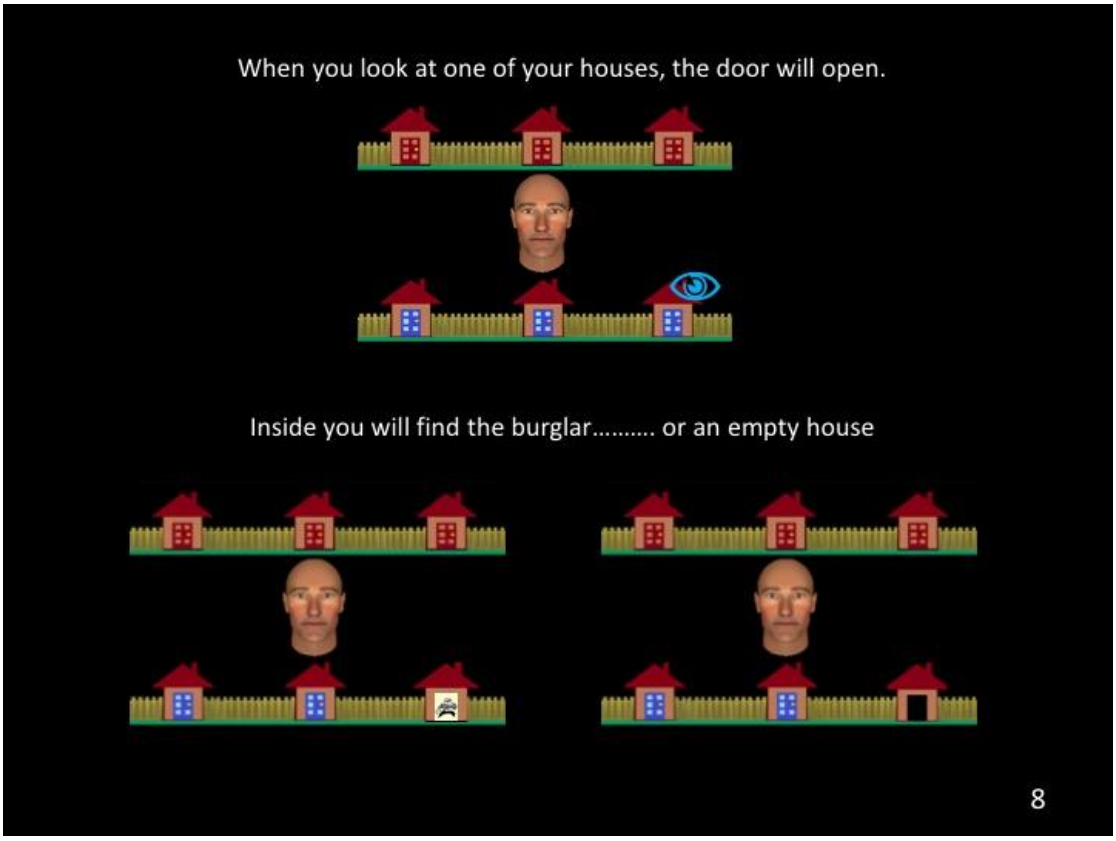
When you look at one of your houses, the door will open.
Inside you will find the burglar………. or an empty house
8

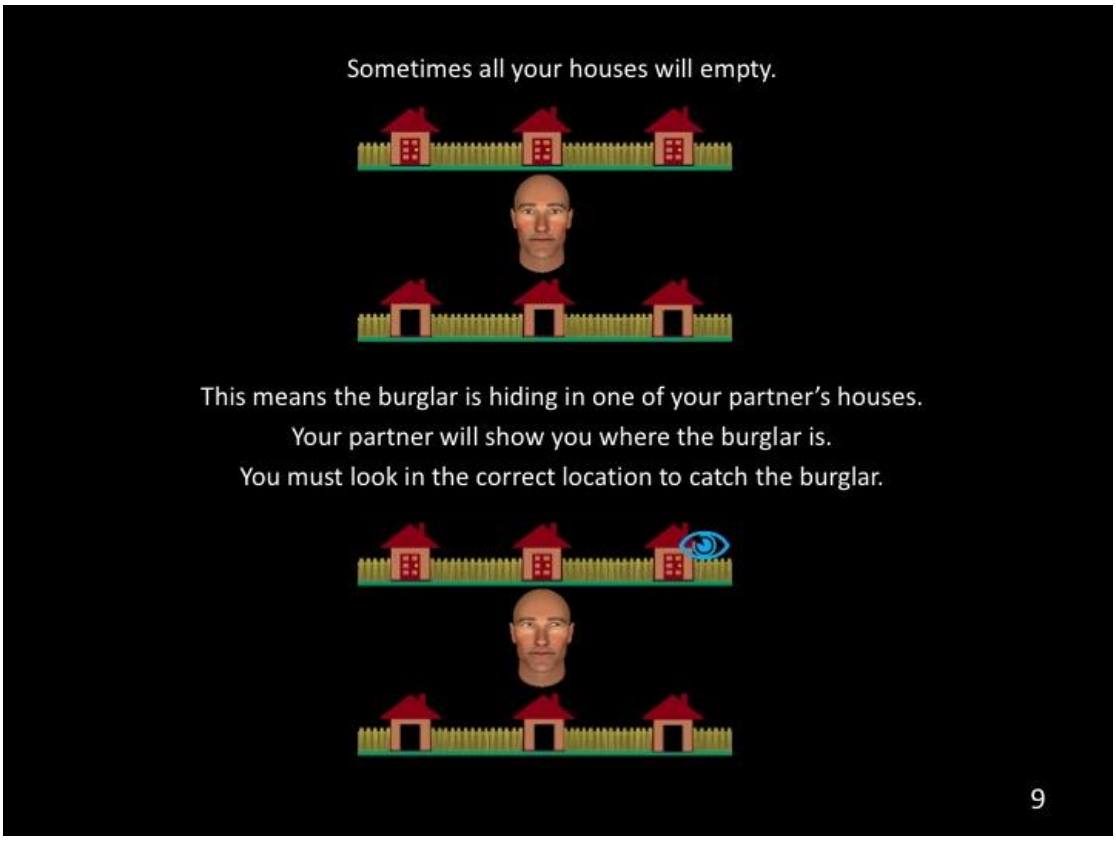
Sometimes all your houses will empty.
This means the burglar is hiding in one of your partner's houses.
Your partner will show you where the burglar is.
You must look in the correct location to catch the burglar.
9

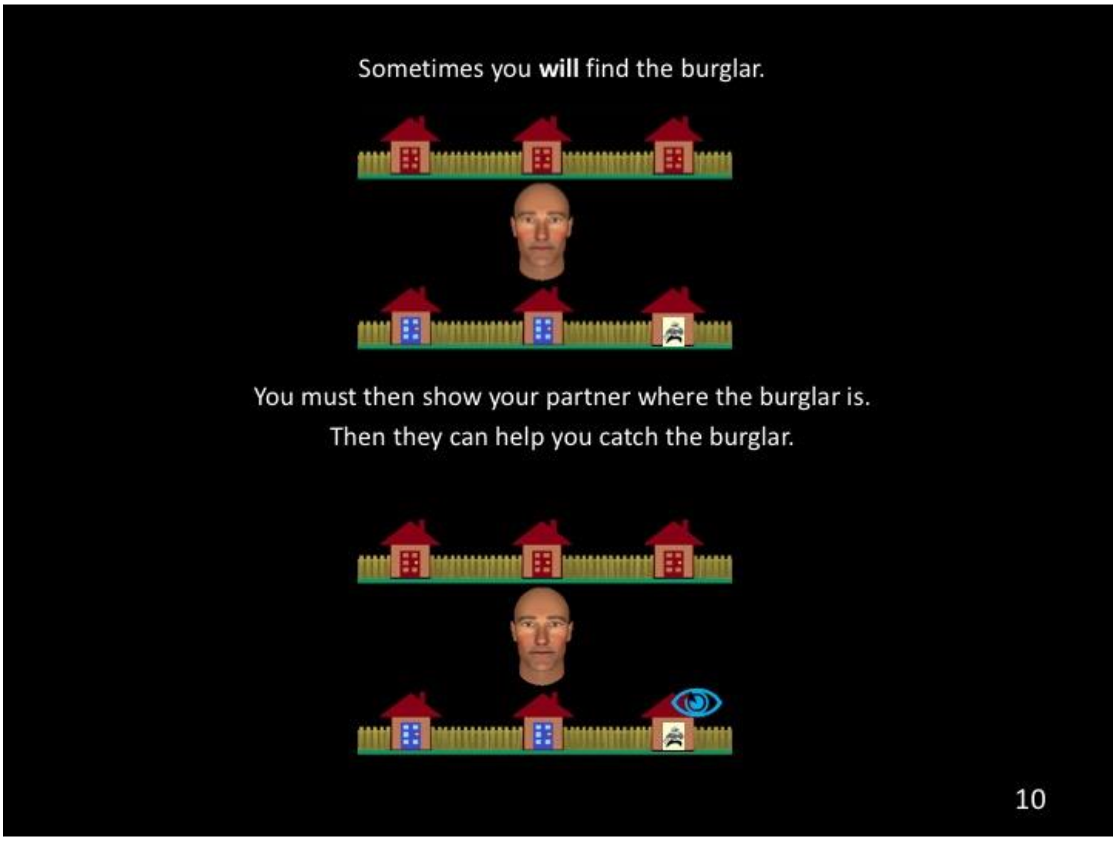
Sometimes you **will** find the burglar.
You must then show your partner where the burglar is.
Then they can help you catch the burglar.
10

You and your partner will also complete the same task on your own.
The virtual interface will be turned off. So the avatar's eyes will close.

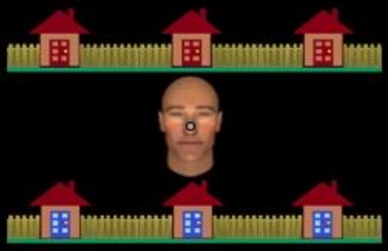

If you don't find the burglar.

1. Look at the dot
2. Then an arrow will guide you

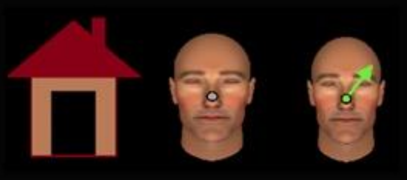

If you DO find the burglar.

1. Look at the dot
2. Wait for the dot to turn green
3. Look back at the burglar's location

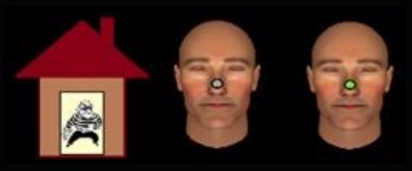

11

When you catch the burglar, he will appear behind bars.

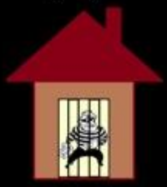

When the burglar escapes, he will appear in red.

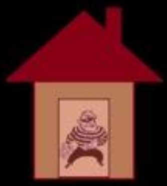

If you do not complete your search, you will see this text.

FAILED SEARCH

12
