# Supplementary material for: Human agency beliefs influence behaviour during virtual social interactions
Source: PeerJ. 2017 Sep 20;5:e3819. doi: 10.7717/peerj.3819 (PMC5610555; doi:10.7717/peerj.3819)

Supplementary Material 2.

Avatar gaze stimuli. (a) top-left (b) top-middle (c) top-right (d) direct gaze (e) eyes closed (f) bottom-left (g) bottom-middle (h) bottom-right.

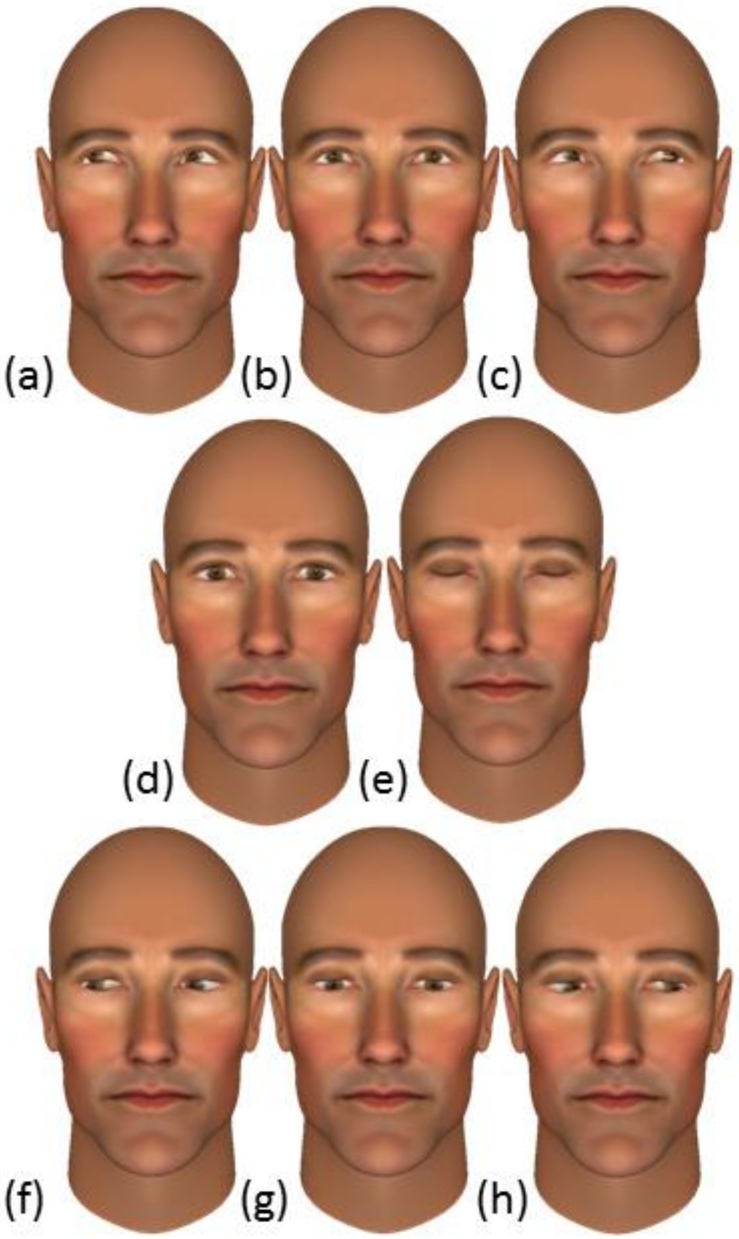

Supplement: Supplemental Information 2 [file peerj-05-3819-s002.pdf]
