# Supplementary material for: Human agency beliefs influence behaviour during virtual social interactions
Source: PeerJ. 2017 Sep 20;5:e3819. doi: 10.7717/peerj.3819 (PMC5610555; doi:10.7717/peerj.3819)
Supplement: Supplemental Information 3 [file peerj-05-3819-s003.html]

Human Agency Beliefs Influence Joint Attention Behaviour


# Human Agency Beliefs Influence Joint Attention Behaviour

#### *Jon Brock*

#### *06/02/2017*

# 1 Background

In this study, we compared the eye-movements of participants as they completed a joint attention game with a virtual partner or avatar. Half of the participants believed that the avatar was controlled by another human. The other half were correctly informed that the avatar was controlled by a computer algorithm.

The code imports trial and interest area reports and combines them into a single dataframe describing each trial. Accuracy and eyetracking data are presented via boxplots. Eyetracking data are analysed via ANOVAs and t-tests. We use non-parmetric stats to explore subject ratings of the task.

## 1.1 Reference

Caruana, N., Spirou, D., & Brock, J. (2017). Human agency beliefs influence joint attention behaviour. Manuscript submitted for publication.

# 2 Load required packages

```
library(reshape2)
library(tables)
library(ez)
library(knitr)
library(plyr)
library(gridExtra)
library(ReporteRs)
library(stringr)
library(dplyr)
library(cowplot)

sessionInfo()
```

```
## R version 3.3.2 (2016-10-31)
## Platform: x86_64-apple-darwin13.4.0 (64-bit)
## Running under: macOS Sierra 10.12.6
## 
## locale:
## [1] en_AU.UTF-8/en_AU.UTF-8/en_AU.UTF-8/C/en_AU.UTF-8/en_AU.UTF-8
## 
## attached base packages:
## [1] stats     graphics  grDevices utils     datasets  methods   base     
## 
## other attached packages:
##  [1] cowplot_0.7.0       dplyr_0.5.0         stringr_1.1.0      
##  [4] ReporteRs_0.8.8     ReporteRsjars_0.0.2 gridExtra_2.2.1    
##  [7] plyr_1.8.4          knitr_1.15.1        ez_4.4-0           
## [10] tables_0.8          Hmisc_4.0-2         ggplot2_2.2.1      
## [13] Formula_1.2-1       survival_2.40-1     lattice_0.20-34    
## [16] reshape2_1.4.2     
## 
## loaded via a namespace (and not attached):
##  [1] Rcpp_0.12.8         png_0.1-7           assertthat_0.1     
##  [4] rprojroot_1.1       digest_0.6.11       mime_0.5           
##  [7] R6_2.2.0            backports_1.0.4     acepack_1.4.1      
## [10] MatrixModels_0.4-1  evaluate_0.10       gdtools_0.1.3      
## [13] lazyeval_0.2.0      minqa_1.2.4         data.table_1.10.4  
## [16] SparseM_1.74        car_2.1-4           nloptr_1.0.4       
## [19] R.utils_2.5.0       R.oo_1.21.0         rpart_4.1-10       
## [22] Matrix_1.2-7.1      checkmate_1.8.2     rmarkdown_1.3      
## [25] splines_3.3.2       lme4_1.1-12         foreign_0.8-67     
## [28] htmlwidgets_0.8     munsell_0.4.3       shiny_1.0.0        
## [31] httpuv_1.3.3        base64enc_0.1-3     mgcv_1.8-15        
## [34] rvg_0.1.2           htmltools_0.3.5     nnet_7.3-12        
## [37] tibble_1.2          htmlTable_1.9       MASS_7.3-45        
## [40] R.methodsS3_1.7.1   grid_3.3.2          DBI_0.6-1          
## [43] xtable_1.8-2        nlme_3.1-128        gtable_0.2.0       
## [46] magrittr_1.5        scales_0.4.1        stringi_1.1.2      
## [49] latticeExtra_0.6-28 xml2_1.1.1          RColorBrewer_1.1-2 
## [52] tools_3.3.2         parallel_3.3.2      pbkrtest_0.4-6     
## [55] yaml_2.1.14         colorspace_1.3-2    cluster_2.0.5      
## [58] rJava_0.9-8         quantreg_5.29
```

# 3 Functions

## 3.1 get\_Boxplot

Draws boxplot with overlaid datapoints

```
get_Boxplot <- function (Data, DV, yMin=0, yMax=1, yBreaks=0.2, yLabel="", PlotTitle="") {
  ggplot(Data, aes_string(x="factor(Condition)", y=DV, fill="Group")) +
    geom_boxplot(outlier.colour=NA) +
    scale_fill_brewer(palette="BuGn") + 
    theme_minimal(base_size = 16, base_family = "") +
    scale_x_discrete("") +
    geom_point(position=position_jitterdodge(dodge.width=0.75, jitter.width=0.3), alpha=0.2, size=3) +
    scale_y_continuous(yLabel,limits=c(yMin,yMax), breaks=seq(yMin,yMax,yBreaks)) +
    ggtitle(PlotTitle) +
    theme(plot.title = element_text(hjust = 0.5))
}
```

## 3.2 get\_legend

Grabs the legend from one of the figures so it can be placed in a multi-panel figure Source: http://www.sthda.com/english/wiki/print.php?id=177

```
get_legend<-function(myggplot){
  tmp <- ggplot_gtable(ggplot_build(myggplot))
  leg <- which(sapply(tmp$grobs, function(x) x$name) == "guide-box")
  legend <- tmp$grobs[[leg]]
  return(legend)
}
```

# 4 Data wrangling

We used Eyelink DataViewer software to export trial and interest area reports as text files.

Extract accuracy data from trial report.

```
  AccData <- read.delim("data/Trial_Report_Accuracy.txt", stringsAsFactors=FALSE)
  Data <- AccData[,c("RECORDING_SESSION_LABEL", "trial_id_num", "INDEX", "ERROR_TYPE")]
  Data$SubNum <- as.numeric(lapply(Data$RECORDING_SESSION_LABEL, function(x) substr(x, 1, 3)))
  Data$UTI <- Data$SubNum*1000 + Data$trial_id_num
```

Extract saccadic reaction times from interest area report and add to trial data.

```
  SRTData <- read.delim("data/IA_Report_RJA_SRT.txt", stringsAsFactors=FALSE)
  SRTData <- SRTData [(SRTData$IA_LABEL=="IA_BURGLAR "),]
  SRTData$IPStartTime <- SRTData$IP_START_TIME - SRTData$TRIAL_START_TIME
  SRTData$IA_FIRST_SACCADE_START_TIME[SRTData$IA_FIRST_SACCADE_START_TIME=="."] <- NA
  SRTData$IA_FIRST_SACCADE_START_TIME <- as.numeric(SRTData$IA_FIRST_SACCADE_START_TIME)
  SRTData$SRT <- SRTData$IA_FIRST_SACCADE_START_TIME - (SRTData$IP_START_TIME - SRTData$TRIAL_START_TIME)
  SRTData$SubNum <- as.numeric(lapply(SRTData$RECORDING_SESSION_LABEL, function(x) substr(x, 1, 3)))
  SRTData$UTI <- SRTData$SubNum*1000 + SRTData$trial_id_num
  Data$FirstSaccadeSRT <- SRTData$SRT[match(Data$UTI, SRTData$UTI)]
```

Extract dwell times from interest area report and add to trial data.

```
  DwellData <- read.delim("data/IA_Report_IJA_Dwell.txt", stringsAsFactors=FALSE) 
  DwellData <- DwellData [(DwellData$IA_LABEL=="IA_BURGLAR "),] 
  DwellData$SubNum <- as.numeric(lapply(DwellData$RECORDING_SESSION_LABEL, function(x) substr(x, 1, 3)))
  DwellData$UTI <- DwellData$SubNum*1000 + DwellData$trial_id_num 
  Data$BurglarDwellTime <- DwellData$IA_DWELL_TIME[match(Data$UTI, DwellData$UTI)]
```

Extract premature saccade data from interest area report and add to trial data.

```
  PremiData <- read.delim("data/IA_Report_IJA_Premi.txt", stringsAsFactors=FALSE)
  PremiData <- PremiData [(PremiData$IA_LABEL=="IA_BURGLAR "),]
  PremiData$PrematureSaccade <- lapply(PremiData$IA_FIXATION_COUNT, function(x) if(x==0){0}else{1})
  PremiData$SubNum <- as.numeric(lapply(PremiData$RECORDING_SESSION_LABEL, function(x) substr(x, 1, 3)))
  PremiData$UTI <- PremiData$SubNum*1000 + PremiData$trial_id_num
  Data$PrematureSaccade <- PremiData$PrematureSaccade[match(Data$UTI, PremiData$UTI)]
  Data$PrematureSaccade <- vapply(Data$PrematureSaccade, paste, collapse = ", ", character(1L)) # For some reason PrematureSaccade is identified as a list. This command "flattens" the list, allowing it to be written to csv.
```

Decode correct response / error type

```
  Data$CorrectResponse <- ifelse(Data$ERROR_TYPE==0,1,0) 
  Data$SearchError <- ifelse(Data$ERROR_TYPE==1,1,0) 
  Data$TimeOut <- ifelse(Data$ERROR_TYPE==2,1,0) 
  Data$LocationError <- ifelse(Data$ERROR_TYPE==3,1,0) 
  Data$RecalibrationError <- ifelse(Data$ERROR_TYPE==4,1,0)
```

Add information about trials.

```
  TrialInfo <- read.csv("data/TrialInfo.csv", stringsAsFactors=FALSE)
  Data$Condition <- TrialInfo$Condition[match(Data$trial_id_num, TrialInfo$trial_id_num)] 
  Data$Interface <- TrialInfo$Interface[match(Data$trial_id_num, TrialInfo$trial_id_num)]
  Data$SubjectRole <- TrialInfo$SubjectRole[match(Data$trial_id_num, TrialInfo$trial_id_num)]
  Data$burglocnum <- TrialInfo$burglocnum[match(Data$trial_id_num, TrialInfo$trial_id_num)]
```

Add information about subjects.

```
  SubjectData <- read.csv("data/SubjectData.csv", stringsAsFactors=FALSE)
  Data$Group <- SubjectData$Group[match(Data$SubNum, SubjectData$SubNum)]
```

Exclude two participants who weren’t deceived.

```
  Data$Exclude <- SubjectData$Exclude[match(Data$SubNum, SubjectData$SubNum)] 
  Data <- Data [(Data$Exclude==0),]
```

Rename dataframe columns for consistency with other datasets.

```
  names(Data)[names(Data)=="trial_id_num"] <- "TrialID" 
  names(Data)[names(Data)=="INDEX"] <- "TrialNum"
```

Remove columns that are now redundant.

```
Data <- subset(Data, select = -c(Exclude, RECORDING_SESSION_LABEL, ERROR_TYPE))
```

Write trial data to CSV file.

```
  write.csv(Data, file="output/data/Data.csv", row.names=FALSE)
```

Identify factors

```
FactorNames <- c("SubNum", "TrialID", "Interface", "SubjectRole", "Group", "Condition")
Data[,FactorNames] <- colwise(as.factor)(Data[,FactorNames])
str(Data)
```

```
## 'data.frame':    10368 obs. of  17 variables:
##  $ TrialID           : Factor w/ 432 levels "1","2","3","4",..: 1 2 3 4 5 6 7 8 9 10 ...
##  $ TrialNum          : int  1 2 3 4 5 6 7 8 9 10 ...
##  $ SubNum            : Factor w/ 48 levels "101","102","103",..: 3 3 3 3 3 3 3 3 3 3 ...
##  $ UTI               : num  103001 103002 103003 103004 103005 ...
##  $ FirstSaccadeSRT   : num  NA NA 486 NA NA 504 627 NA NA NA ...
##  $ BurglarDwellTime  : int  0 2956 0 1256 698 0 0 636 624 1938 ...
##  $ PrematureSaccade  : chr  "0" "1" "0" "1" ...
##  $ CorrectResponse   : num  1 1 1 1 1 1 1 0 1 1 ...
##  $ SearchError       : num  0 0 0 0 0 0 0 0 0 0 ...
##  $ TimeOut           : num  0 0 0 0 0 0 0 1 0 0 ...
##  $ LocationError     : num  0 0 0 0 0 0 0 0 0 0 ...
##  $ RecalibrationError: num  0 0 0 0 0 0 0 0 0 0 ...
##  $ Condition         : Factor w/ 4 levels "IJA","IJAc","RJA",..: 4 2 4 2 2 4 3 1 1 1 ...
##  $ Interface         : Factor w/ 2 levels "Control","Test": 1 1 1 1 1 1 2 2 2 2 ...
##  $ SubjectRole       : Factor w/ 2 levels "Initiate","Respond": 2 1 2 1 1 2 2 1 1 1 ...
##  $ burglocnum        : int  2 5 2 5 4 1 1 5 5 6 ...
##  $ Group             : Factor w/ 2 levels "Computer","Human": 1 1 1 1 1 1 1 1 1 1 ...
```

# 5 Accuracy

Screen data, aggregate, and re-order conditions.

```
ScreenedData <- filter(Data, RecalibrationError==0, SearchError==0, !is.na(CorrectResponse))
BySubjectsData <- ddply(ScreenedData, .(Group, SubNum, Condition, SubjectRole, Interface), summarise, ProportionCorrect = mean(CorrectResponse))
BySubjectsData$Condition <- factor(BySubjectsData$Condition, c("RJA", "RJAc", "IJA", "IJAc"))
```

Create boxplot

```
Acc_Boxplot <- get_Boxplot(BySubjectsData, "ProportionCorrect", yMin=0, yMax=1, yBreaks=0.2, yLabel="Proportion Correct", PlotTitle = "Accuracy")
Acc_Boxplot
```

Three-way ANOVA on ArcSin-transformed proportion of correct responses. This shows better performance for Initiating than Responding and better performance for the Control condition than the Social condition. There’s also an interaction reflecting the fact that errors are largest for the RJA condition than the other three conditions. There is no effect of Group and there are no interactions involving Group. In other words, there is no evidence that believing you are interacting with a Human vs a Computer affects your accuracy. However, the ceiling effects mean that all of these conclusions should be treated with extreme caution.

```
BySubjectsData$AsinCorrect <- asin(BySubjectsData$ProportionCorrect)
kable(ezANOVA(BySubjectsData, dv=AsinCorrect, wid=SubNum, within=.(Interface, SubjectRole), between=Group, type = 3), row.names=FALSE, caption="ANOVA: Accuracy", digits=3)
```

ANOVA: Accuracy

| | Effect | DFn | DFd | F | p | p<.05 | ges | | --- | --- | --- | --- | --- | --- | --- | | Group | 1 | 46 | 3.212 | 0.080 |  | 0.017 | | Interface | 1 | 46 | 43.863 | 0.000 | \* | 0.142 | | SubjectRole | 1 | 46 | 95.869 | 0.000 | \* | 0.427 | | Group:Interface | 1 | 46 | 0.384 | 0.539 |  | 0.001 | | Group:SubjectRole | 1 | 46 | 0.225 | 0.637 |  | 0.002 | | Interface:SubjectRole | 1 | 46 | 36.691 | 0.000 | \* | 0.150 | | Group:Interface:SubjectRole | 1 | 46 | 0.586 | 0.448 |  | 0.003 | |

Given that errors occurred primarily in the RJA condition, one possibility is that participants had difficulty detecting eye gaze to particular locations or discriminating between certain locations. However, breaking response accuracy down by location showed no discernable pattern.

NB: This was an exploratory analysis conducted at the request of a reviewer and is not reported in the paper.

```
ScreenedData <- filter(Data, Condition=="RJA", RecalibrationError==0, SearchError==0, !is.na(CorrectResponse))
BySubjectsData <- ddply(ScreenedData, .(Group, SubNum, Condition, burglocnum), summarise, ProportionCorrect = mean(CorrectResponse))

get_Boxplot(BySubjectsData, "ProportionCorrect", yMin=0, yMax=1, yBreaks=0.2, yLabel="Proportion Correct", PlotTitle = "Accuracy by location") +
  facet_wrap(~burglocnum, ncol=3)
```

```
BySubjectsData$AsinCorrect <- asin(BySubjectsData$ProportionCorrect)
kable(ezANOVA(BySubjectsData, dv=AsinCorrect, wid=SubNum, within=.(burglocnum), between=Group, type = 3), row.names=FALSE, caption="ANOVA: Accuracy by Location for RJA", digits=3)
```

ANOVA: Accuracy by Location for RJA

| | Effect | DFn | DFd | F | p | p<.05 | ges | | --- | --- | --- | --- | --- | --- | --- | | Group | 1 | 46 | 1.737 | 0.194 |  | 0.016 | | burglocnum | 1 | 46 | 1.543 | 0.220 |  | 0.019 | | Group:burglocnum | 1 | 46 | 0.066 | 0.799 |  | 0.001 | |

# 6 Saccadic reaction times

Screen data, aggregate.

```
ScreenedData <- filter(Data, SubjectRole=="Respond", CorrectResponse==1, FirstSaccadeSRT>=150, FirstSaccadeSRT<=3000, !is.na(FirstSaccadeSRT))
BySubjectsData <- ddply(ScreenedData, .(Group, SubNum, Condition), summarise, FirstSaccadeSRT = mean(FirstSaccadeSRT))
```

Draw boxplot

```
RJA_SRT_Boxplot <- get_Boxplot(BySubjectsData, "FirstSaccadeSRT", yMin=0, yMax=1200, yBreaks=200, yLabel="Duration / ms", PlotTitle = "Saccadic Reaction Time")
RJA_SRT_Boxplot
```

Two way ANOVA shows participants are faster to respond to the arrow cue (RJAc) than eye gaze cue (RJA). Those in the Human group are significantly faster to respond than those in the Computer group. However, there is no interaction.

```
kable(ezANOVA(BySubjectsData, dv = FirstSaccadeSRT, wid = SubNum, within = .(Condition), between = Group, type = 3), row.names=FALSE, caption="First saccade reaction time", digits=3)
```

First saccade reaction time

| | Effect | DFn | DFd | F | p | p<.05 | ges | | --- | --- | --- | --- | --- | --- | --- | | Group | 1 | 46 | 5.710 | 0.021 | \* | 0.075 | | Condition | 1 | 46 | 264.629 | 0.000 | \* | 0.664 | | Group:Condition | 1 | 46 | 2.340 | 0.133 |  | 0.017 | |

# 7 Burglar dwell times

Screen data and aggregate

```
ScreenedData <- filter(Data, SubjectRole=="Initiate", CorrectResponse==1, BurglarDwellTime>=150, BurglarDwellTime<=3000, !is.na(BurglarDwellTime))
BySubjectsData <- ddply(ScreenedData, .(Group, SubNum, Condition), summarise, BurglarDwellTime = mean(BurglarDwellTime))
```

Draw boxplot

```
IJA_Dwell_Boxplot <- get_Boxplot (BySubjectsData, "BurglarDwellTime", yMin=0, yMax=1800, yBreaks=200, yLabel="Duration / ms", PlotTitle = "Dwell Time on Burglar")
IJA_Dwell_Boxplot
```

Two-way ANOVA shows that participants are slower to saccade back to the avatar in the IJA condition compared to the control (IJAc) condition. There is also a significant interaction.

```
kable(ezANOVA(BySubjectsData, dv = BurglarDwellTime, wid = SubNum, within = .(Condition), between = Group, type = 3), row.names=FALSE, caption="ANOVA: Burglar Dwell Time", digits=3)
```

ANOVA: Burglar Dwell Time

| | Effect | DFn | DFd | F | p | p<.05 | ges | | --- | --- | --- | --- | --- | --- | --- | | Group | 1 | 46 | 0.055 | 0.816 |  | 0.001 | | Condition | 1 | 46 | 24.361 | 0.000 | \* | 0.043 | | Group:Condition | 1 | 46 | 14.723 | 0.000 | \* | 0.026 | |

This interaction arises because the difference between IJA and IJAc is only apparent when participants think they are interacting with another Human.

```
TTest.Dwell <- data.frame(Measure=c("Dwell Time", "Dwell Time"),
                       Group=c("Computer", "Human"),
                       df=numeric(2),
                       t=numeric(2),
                       p=numeric(2))

for (i in 1:2) {
  Group <- TTest.Dwell$Group[i]
  TTest <- t.test(BurglarDwellTime ~ Condition, BySubjectsData[(BySubjectsData$Group==Group),], paired=TRUE)
  TTest.Dwell$df[i] <- TTest$parameter
  TTest.Dwell$t[i] <- TTest$statistic
  TTest.Dwell$p[i] <- TTest$p.value
}
kable(TTest.Dwell, row.names=FALSE, caption="T-tests: Effect of Condition on Dwell Times", digits=3)
```

T-tests: Effect of Condition on Dwell Times

| Measure | Group | df | t | p |
| --- | --- | --- | --- | --- |
| Dwell Time | Computer | 23 | 0.888 | 0.383 |
| Dwell Time | Human | 23 | 5.581 | 0.000 |

## 7.1 Premature saccades

Screen and aggregate data.

```
ScreenedData <- filter(Data, SubjectRole=="Initiate", RecalibrationError==0, SearchError==0, !is.na(PrematureSaccade))
ScreenedData$PrematureSaccade <- as.numeric(ScreenedData$PrematureSaccade)
BySubjectsData <- ddply(ScreenedData, .(Group, SubNum, Condition), summarise, PrematureSaccades = mean(PrematureSaccade))
```

Draw boxplot

```
IJA_Premi_Boxplot <- get_Boxplot (BySubjectsData, "PrematureSaccades", yMin=0, yMax=1, yBreaks=0.2, yLabel="Proportion of Trials", PlotTitle = "Premature Saccades")
IJA_Premi_Boxplot
```

ANOVA shows that there are more premature saccades for IJA than IJAc. However, there is a significant interaction with Group (Human vs Computer)

```
kable(ezANOVA(BySubjectsData, dv = PrematureSaccades, wid = SubNum, within = .(Condition), between = Group, type = 3), row.names=FALSE, caption="PrematureSaccade", digits=3)
```

```
## Warning: You have removed one or more levels from variable "Condition".
## Refactoring for ANOVA.
```

PrematureSaccade

| | Effect | DFn | DFd | F | p | p<.05 | ges | | --- | --- | --- | --- | --- | --- | --- | | Group | 1 | 46 | 3.780 | 0.058 |  | 0.062 | | Condition | 1 | 46 | 38.494 | 0.000 | \* | 0.141 | | Group:Condition | 1 | 46 | 13.788 | 0.001 | \* | 0.055 | |

This interaction arises because the effect of Condition is larger for the Human group than the Computer group.

```
TTest.Premi <- data.frame(Measure=c("Premature Saccades", "Premature Saccades"),
                       Group=c("Computer", "Human"),
                       df=numeric(2),
                       t=numeric(2),
                       p=numeric(2))

for (i in 1:2) {
  Group <- TTest.Premi$Group[i]
  TTest <- t.test(PrematureSaccades ~ Condition, BySubjectsData[(BySubjectsData$Group==Group),], paired=TRUE)
  TTest.Premi$df[i] <- TTest$parameter
  TTest.Premi$t[i] <- TTest$statistic
  TTest.Premi$p[i] <- TTest$p.value
}
kable(TTest.Premi, row.names=FALSE, caption="T-tests: Effect of Condition on Premature Saccades", digits=3)
```

T-tests: Effect of Condition on Premature Saccades

| Measure | Group | df | t | p |
| --- | --- | --- | --- | --- |
| Premature Saccades | Computer | 23 | 2.109 | 0.046 |
| Premature Saccades | Human | 23 | 6.145 | 0.000 |

We get a similar pattern of results if we take the ArcSin transformation of the proportion of premature saccades.

```
BySubjectsData$AsinPremature <- asin(BySubjectsData$PrematureSaccades)

kable(ezANOVA(BySubjectsData, dv = AsinPremature, wid = SubNum, within = .(Condition), between = Group, type = 3), row.names=FALSE, caption="PrematureSaccade", digits=3)
```

```
## Warning: You have removed one or more levels from variable "Condition".
## Refactoring for ANOVA.
```

PrematureSaccade

| | Effect | DFn | DFd | F | p | p<.05 | ges | | --- | --- | --- | --- | --- | --- | --- | | Group | 1 | 46 | 3.882 | 0.055 |  | 0.064 | | Condition | 1 | 46 | 36.606 | 0.000 | \* | 0.133 | | Group:Condition | 1 | 46 | 14.063 | 0.000 | \* | 0.056 | |

```
TTest.Premi <- data.frame(Measure=c("Premature Saccades", "Premature Saccades"),
                       Group=c("Computer", "Human"),
                       df=numeric(2),
                       t=numeric(2),
                       p=numeric(2))

for (i in 1:2) {
  Group <- TTest.Premi$Group[i]
  TTest <- t.test(AsinPremature ~ Condition, BySubjectsData[(BySubjectsData$Group==Group),], paired=TRUE)
  TTest.Premi$df[i] <- TTest$parameter
  TTest.Premi$t[i] <- TTest$statistic
  TTest.Premi$p[i] <- TTest$p.value
}
kable(TTest.Premi, row.names=FALSE, caption="T-tests: Effect of Condition on Premature Saccades", digits=3)
```

T-tests: Effect of Condition on Premature Saccades

| Measure | Group | df | t | p |
| --- | --- | --- | --- | --- |
| Premature Saccades | Computer | 23 | 2.104 | 0.047 |
| Premature Saccades | Human | 23 | 5.852 | 0.000 |

# 8 Ratings data

```
RatingsData <- subset(SubjectData[(SubjectData$Exclude==0),], select = -c(Exclude, Sex, Age, EdHAnd, Block.1, Block.2, ET_Accuracy) )

RatLong <- melt(RatingsData, id=c("Group", "SubNum"), variable.name="Measure", value.name="Rating")

RatLong$Question <- as.factor(str_split_fixed(RatLong$Measure, "_", 2)[,1])
RatLong$Condition <- as.factor(str_split_fixed(RatLong$Measure, "_", 2)[,2])

RatLong$Question <- mapvalues(RatLong$Question, from = c("PreferVirtual", "PreferAlone", "Feel", "Behave", "Appear"), to = c("Prefer Virtual", "Prefer Alone", "Feel Humanlike", "Behave Humanlike", "Appear Humanlike"))

QuestionList <- c("Prefer Virtual", "Prefer Alone", "Behave Humanlike", "Appear Humanlike", "Feel Humanlike", "Cooperative", "Pleasant","Intuitive","Natural","Difficult")

RatingsBoxplot <- ggplot(RatLong[(RatLong$Condition!="Arrow"),], aes(x=factor(Question), y=Rating, fill=Group)) +
    geom_boxplot(outlier.colour=NA) +
    theme_minimal(base_size = 16, base_family = "") +
    xlim(QuestionList) +
    xlab("Question") +
    scale_y_continuous("Rating",limits=c(1,10), breaks=seq(1,10,1)) +
    scale_fill_brewer(palette="BuGn")+
    coord_flip()
RatingsBoxplot
```

```
Wilcoxon.DF <- data.frame(Question=rev(QuestionList),
                          W=numeric(length(QuestionList)),
                          p=numeric(length(QuestionList)))

for (i in 1:length(QuestionList)) {
  QuestionData <- RatLong[(RatLong$Question==Wilcoxon.DF$Question[i]) & (RatLong$Condition=="Alan" | RatLong$Condition=="Both"),]
  Wilcox <- wilcox.test(Rating ~ Group, data = QuestionData, paired=FALSE, exact=FALSE)
  Wilcoxon.DF$W[i] <- Wilcox$statistic
  Wilcoxon.DF$p[i] <- Wilcox$p.value
}

kable(Wilcoxon.DF, row.names=FALSE, caption="Wilcoxon rank sum test with continuity correction", digits=3)
```

Wilcoxon rank sum test with continuity correction

| Question | W | p |
| --- | --- | --- |
| Difficult | 346.5 | 0.209 |
| Natural | 216.5 | 0.139 |
| Intuitive | 384.5 | 0.045 |
| Pleasant | 188.5 | 0.038 |
| Cooperative | 193.0 | 0.039 |
| Feel Humanlike | 206.5 | 0.090 |
| Appear Humanlike | 284.5 | 0.950 |
| Behave Humanlike | 214.0 | 0.124 |
| Prefer Alone | 358.5 | 0.142 |
| Prefer Virtual | 299.5 | 0.817 |

# 9 Output

## 9.1 Tables

```
TTest.DF <- rbind(TTest.Dwell, TTest.Premi)
doc <- docx()
doc <- addTitle(doc, "Table 2: ", level=2)
doc <- addFlexTable( doc, vanilla.table(TTest.DF))
writeDoc(doc, file = "output/doc/Tables.docx")
```

## 9.2 Figures

```
legend <- get_legend(Acc_Boxplot)

Fig2 <- plot_grid(Acc_Boxplot + theme(legend.position="none"), legend, nrow=1, rel_widths = c(1,0.3), vjust = -1, scale = 0.85)
png(filename="output/figures/Fig2.png", width=600, height=500)
Fig2
dev.off()
```

```
## quartz_off_screen 
##                 2
```

```
knitr::include_graphics("output/figures/Fig2.png")
```

```
legend <- get_legend(RJA_SRT_Boxplot)
Fig3 <- plot_grid(RJA_SRT_Boxplot + theme(legend.position="none"), IJA_Dwell_Boxplot + theme(legend.position="none"), IJA_Premi_Boxplot + theme(legend.position="none"), legend, nrow=1, labels = c('A', 'B', 'C'), label_size = 14, rel_widths = c(1,1,1,0.3), vjust = -1, scale = 0.85)

png(filename="output/figures/Fig3.png", width=1200, height=500)
Fig3
dev.off()
```

```
## quartz_off_screen 
##                 2
```

```
knitr::include_graphics("output/figures/Fig3.png")
```

```
#grid.arrange (RJA_SRT_Boxplot + theme(legend.position="none"), IJA_Dwell_Boxplot + theme(legend.position="none"), IJA_Premi_Boxplot + theme(legend.position="none"), legend, ncol=4, nrow =1, widths=c(2.3, 2.3, 2.3, 0.8), heights=c(2.3))


png(filename="output/figures/Fig4.png", width=600, height=600)
RatingsBoxplot
dev.off()
```

```
## quartz_off_screen 
##                 2
```

```
knitr::include_graphics("output/figures/Fig4.png")
```
